# Characteristics and outcomes of culture-negative versus culture-positive severe sepsis

**DOI:** 10.1186/cc12896

**Published:** 2013-09-12

**Authors:** Jason Phua, Wang Jee Ngerng, Kay Choong See, Chee Kiang Tay, Timothy Kiong, Hui Fang Lim, Mei Ying Chew, Hwee Seng Yip, Adeline Tan, Haji Jamil Khalizah, Rolando Capistrano, Kang Hoe Lee, Amartya Mukhopadhyay

**Affiliations:** 1 Division of Respiratory and Critical Care Medicine, University Medicine Cluster, National University Hospital, National University Health System Tower Block Level 10, 1E Kent Ridge Road, Singapore 119228; 2 Department of Medicine, Yong Loo Lin School of Medicine, National University of Singapore, National University Health System Tower Block Level 10, 1E Kent Ridge Road, Singapore 119228; 3 Faculty of Medicine, Suite H 2743, 300 Prince Philip Drive, Memorial University of Newfoundland, St. John's NL, A1B 3V6, Canada; 4 Department of Medicine, Alexandra Hospital (Jurong Health Services), 378 Alexandra Road, Singapore 159964; 5 RIPAS Hospital, Jalan Putera Al-Muhtadee Billah, Bandar Seri Begawan, BA 1710, Brunei Darussalam; 6 Asian Centre for Liver Diseases and Transplantation, Gleneagles Hospital, Annexe Block, 02-37, 6A Napier Road, Singapore 258500

## Abstract

**Introduction:**

Culture-negative sepsis is a common but relatively understudied condition. The aim of this study was to compare the characteristics and outcomes of culture-negative versus culture-positive severe sepsis.

**Methods:**

This was a prospective observational cohort study of 1001 patients who were admitted to the medical intensive care unit (ICU) of a university hospital from 2004 to 2009 with severe sepsis. Patients with documented fungal, viral, and parasitic infections were excluded.

**Results:**

There were 415 culture-negative patients (41.5%) and 586 culture-positive patients (58.5%). Gram-positive bacteria were isolated in 257 patients, and gram-negative bacteria in 390 patients. Culture-negative patients were more often women and had fewer comorbidities, less tachycardia, higher blood pressure, lower procalcitonin levels, lower Acute Physiology and Chronic Health Evaluation II (median 25.0 (interquartile range 19.0 to 32.0) versus 27.0 (21.0 to 33.0), *P *= 0.001) and Sequential Organ Failure Assessment scores, less cardiovascular, central nervous system, and coagulation failures, and less need for vasoactive agents than culture-positive patients. The lungs were a more common site of infection, while urinary tract, soft tissue and skin infections, infective endocarditis and primary bacteremia were less common in culture-negative than in culture-positive patients. Culture-negative patients had a shorter duration of hospital stay (12 days (7.0 to 21.0) versus 15.0 (7.0 to27.0), *P *= 0.02) and lower ICU mortality than culture-positive patients. Hospital mortality was lower in the culture-negative group (35.9%) than in the culture-positive group (44.0%, *P *= 0.01), the culture-positive subgroup, which received early appropriate antibiotics (41.9%, *P *= 0.11), and the culture-positive subgroup, which did not (55.5%, *P *< 0.001). After adjusting for covariates, culture positivity was not independently associated with mortality on multivariable analysis.

**Conclusions:**

Significant differences between culture-negative and culture-positive sepsis are identified, with the former group having fewer comorbidities, milder severity of illness, shorter hospitalizations, and lower mortality.

## Introduction

Severe sepsis is a major cause of morbidity and mortality in both developed and developing countries [1]. Mortality rates remain high at 30% and rise to 60% in the presence of septic shock despite significant advancement in treatment modalities [2]. Bacteria are by far the most common causative microorganisms in sepsis, and cultures are positive in about 50% of cases [3]. Failure to administer antibiotics to which the pathogens are susceptible is associated with increased mortality [4]. Thus, early broad-spectrum antibacterial agents are recommended as a means to improve survival [5].

Less is known though about the other half of the equation: sepsis for which etiologic agents are not found. It is commonly thought that cultures may lack the sensitivity to detect all infecting bacteria [6]. Beyond this, and aside from data from a few multicenter epidemiological studies, which suggest that severity of illness and mortality are not significantly affected by microbiological documentation in sepsis [7-12], the medical literature is surprisingly devoid of information about patients with culture-negative sepsis.

The aim of our study was hence to compare the characteristics and outcomes of culture-negative versus culture-positive severe sepsis.

## Materials and methods

### Study design

This was a prospective observational cohort study conducted in the medical intensive care unit (ICU) of our university hospital. The study, being non-interventional, was approved by our institutional review board, the National Healthcare Group's Domain Specific Review Board, with a waiver of informed consent.

### Inclusion criteria

We included all patients who were admitted to our ICU from 2004 to 2009 for severe sepsis, which was defined according to the 1992 American College of Chest Physicians (ACCP)/Society of Critical Care Medicine (SCCM) Consensus Conference criteria, that is, sepsis with at least one organ dysfunction [13]. The diagnosis of sepsis required the presence of the systemic inflammatory response syndrome due to infection.

### Exclusion criteria

As we were interested in comparing acute culture-negative sepsis with culture-positive bacterial sepsis, we excluded patients with microbiogically proven fungal, viral, and parasitic infections, and tuberculosis. We only recorded the first ICU admission and excluded readmissions.

### Diagnosis of infection

Infection was diagnosed clinically by the managing physicians. From the year 2005 onward, reference was made to the International Sepsis Forum Consensus Conference guidelines on definitions of infections where appropriate [14]. Briefly, the diagnosis of pneumonia required a radiographic infiltrate plus a high clinical suspicion, including fever or hypothermia, leukocytosis or leukopenia, and purulent respiratory secretions. Patients were deemed culture-positive if etiologic agents were recovered from blood or pleural fluid, or if semi-quantitative cultures of sputum, blind endotracheal aspirates, or bronchoalveolar lavage found moderate to heavy growths of bacteria with few epithelial cells seen on Gram stain examination (≤10 per high power field). Intra-abdominal infections included but were not limited to intra-abdominal abscesses, peritonitis, biliary tract infections, pancreatic infections, enteritis, and colitis. Urinary tract infection was diagnosed through typical signs and symptoms including fever, urgency, frequency, dysuria, pyuria, hematuria, positive Gram stain, pus, and suggestive imaging. Urine cultures were deemed positive with the isolation of >10^5 ^colony forming units (cfu)/mL of microorganisms (or 10^3 ^cfu/mL in catheterized patients). Soft tissue and skin infections included surgical site infections, cellulitis, and necrotizing fasciitis. Infective endocarditis was diagnosed based on the revised Duke criteria. When diagnosing bacteremia, common skin contaminants like coagulase-negative staphylococci, *Bacillus *species, *Corynebacterium *species, micrococci, and *Propionibacterium *species were disregarded unless they were deemed clinically significant by the managing physicians or cultured from two or more blood cultures. Primary bacteremia was diagnosed when the microorganism cultured was not related to an infection at another site. Catheter-related sepsis is the only infection for which microbiological confirmation was mandated by the International Sepsis Forum [14]. For this study, the diagnosis of culture-positive catheter-related sepsis required a positive peripheral blood culture, while the diagnosis of culture-negative catheter-related sepsis was made clinically in the presence of pus or cellulitis at the exit site or tunnel tract infection.

### Clinical management

Patient care in the ICU was left to the discretion of the managing physicians, who were encouraged to follow the Surviving Sepsis Campaign guidelines after they were published in 2004 [15]. While the treatments were not protocolized, broadly, they involved aggressive fluid resuscitation and vasopressors, with hemodynamic information obtained via lactate and N-terminal B-type natriuretic peptide measurements, transthoracic echocardiography, arterial pressure waveform analyses, and transpulmonary thermodilution when indicated. Early intubation was advocated for respiratory failure. Blood cultures were obtained early, with 20 mL of blood distributed equally for each set of aerobic and anaerobic media, while cultures of other sites were performed depending on the source of infection. Empiric broad spectrum antibiotics were chosen based on the suspected infection and optimized and/or de-escalated according to the culture results.

### Data collection

A research coordinator (WJN) prospectively entered the data into a Computerized Clinical Research Database under the close supervision of the principal investigator (JP). Patients were followed till discharge from or death in the hospital. The inputted data and electronic case records for all patients were then retrospectively reviewed by the co-investigators. Data including statistical outliers that might represent entry errors were verified and corrected in cases of inconsistency.

Data collected were baseline variables on entry to the ICU including patient demographics, source and time of admission, comorbidities, vital signs and blood investigations (white blood cell count, procalcitonin, and C-reactive protein where available), and variables on the first day of ICU admission including the Acute Physiology and Chronic Health Evaluation (APACHE) II and the corresponding Acute Physiology Score, the Sequential Organ Failure Assessment (SOFA) score, and treatment provided (vasoactive agents, mechanical ventilation, renal replacement therapy, and glucocorticoids for septic shock). We defined organ failures as a SOFA score of >2 for the organs concerned [11]. We documented the site(s) of infection based on the clinical impression of the managing physicians. To ensure that any bacteria isolated were the cause of severe sepsis that resulted in ICU admission, we recorded results of all bacteria cultures collected within the two days before and the two days after admission, unless they were deemed to be colonizers or contaminants by the managing physicians; in the latter cases, adjudication was provided by the principal investigator (JP). Bacteria isolated more than two days before ICU admission were only logged if they were judged to have led to the clinical deterioration by the managing physicians. We charted all antibiotics administered on the day of ICU admission and defined the initial antimicrobial therapy as appropriate if positive cultures were susceptible to any of these antibiotics or if all cultures were negative, and as inappropriate if positive cultures were not susceptible to all of these antibiotics [4].

The primary outcome variable was hospital mortality, while the secondary outcome variables were ICU mortality, duration of mechanical ventilation, ICU stay, and hospital stay.

### Statistical analyses

We classified the patients into two groups depending on whether bacteria which caused the severe sepsis were found (culture-positive) or not found (culture-negative). We expressed categorical variables as number (percentage). After using the Kolmogorov-Smirnov test and examining histograms to verify if normality and homogeneity assumptions were satisfied, we expressed normally distributed numerical variables as mean (95% confidence interval (CI)) and other numerical variables as median (interquartile range). We compared categorical variables using the χ^2 ^test or Fisher exact test, normally distributed quantitative variables with the *t *test, and other quantitative variables with the Mann-Whitney *U *test. We used the Bonferroni correction for pairwise comparisons.

To identify the independent predictors of hospital mortality, in addition to univariable analyses, we performed a multivariable logistic regression analysis using a model that included variables which could potentially affect survival, that is, all recorded variables at baseline and on day one in the ICU, the site of infection, whether the patients were culture-negative or culture-positive, whether the initial antimicrobial therapy was appropriate or inappropriate, and whether bacteremia was absent or present. We looked for multicollinearity, and assessed model fit using the Hosmer-Lemeshow goodness-of-fit test. To identify the specific bacteria that were independently associated with mortality, we repeated the regression analysis after substituting the five commonest Gram-negative microorganisms and the five commonest Gram-positive microorganisms for the broad groups of culture negativity versus culture positivity as covariates into the model. We considered a *P *value of < 0.05 significant and used IBM SPSS version 20.0 (IBM Corp, Armonk, NY, USA).

## Results

The study included 415 culture-negative patients (41.5%) and 586 culture-positive patients (58.5%) who were admitted to our ICU for severe sepsis. Table 1 describes their characteristics at baseline and on day one of the ICU stay. Compared to culture-positive patients, culture-negative patients were more likely to be women, have fewer comorbid conditions, less tachycardia, higher blood pressure, lower procalcitonin levels, lower APACHE II and SOFA scores, and less cardiovascular, central nervous system, and coagulation failures. Culture-negative patients were less likely to be treated with vasoactive agents on the first day of ICU stay.

**Table 1:** Characteristics at baseline and on day one of intensive care unit admission.

	Culture-negative patients (*n *= 415)	Culture-positive patients (*n *= 586)	*P *value
Demographics			
Age, years	62.0 (50.0-74.0)	64.0 (50.1-74.0)	0.62
Men	236 (56.9)	375 (64.0)	0.02
Race			0.09
Chinese	229 (55.2)	358 (61.1)	
Malay	106 (25.5)	120 (20.5)	
Indian	56 (13.5)	64 (10.9)	
Others	24 (5.8)	44 (7.5)	
Source of admission			0.49
Emergency department	195 (47.0)	257 (43.9)	
Hospital floor	195 (47.0)	293 (50.0)	
Other ICU	25 (6.0)	34 (5.8)	
Operating room/recovery	0 (0)	2 (0.3)	
Time of ICU admission			
Day of hospital admission	0 (0-2.00)	0 (0-2.00)	0.38
Office hour admission	166 (40.0)	260 (44.4)	0.17
Comorbid conditions			
Diabetes mellitus	141 (34.0)	234 (39.9)	0.06
Chronic heart failure	22 (5.3)	40 (6.8)	0.32
Chronic kidney disease	48 (11.6)	96 (16.4)	0.03
COPD	24 (5.8)	38 (6.5)	0.65
Cancer	29 (7.0)	55 (9.4)	0.18
Hematological malignancy	9 (2.2)	24 (4.1)	0.09
No. of comorbid conditions^a^			0.004
0	214 (51.6)	241 (41.1)	
1	136 (32.8)	225 (38.4)	
2	59 (14.2)	98 (16.7)	
≥3	6 (1.4)	22 (3.8)	
Vital signs upon ICU admission			
Temperature, °C	37.0 (36.0-38.0)	37 (36.2-38.0)	0.29
Heart rate, /min	107.3 ± 27.5	113.4 ± 25.9	<0.001
Mean blood pressure, mm Hg	78.0 (64.0-96.0)	75.0 (62.0-91.0)	0.01
Respiratory rate, /min	24.0 (20.0-31.0)	25.0 (20.0-31.0)	0.84
Investigations^b^			
White blood cell, 10^9^/L	13.27 (9.18-19.47)	13.44 (6.58-19.65)	0.19
Procalcitonin, ng/mL	3.95 (0.90-17.28)	16.40 (2.74-47.03)	<0.001
C-reactive protein, mg/L	47.50 (14.05-191.50)	51.50 (11.63-181.50)	0.53
Severity scores			
APACHE II	25.0 (19.0-32.0)	27.0 (21.0-33.0)	0.001
Acute Physiology Score	20.0 (14.0-26.0)	22.0 (16.8-28.3)	<0.001
SOFA	9.0 (6.0-12.0)	10.0 (8.0-13.0)	<0.001
Organ failure^c^			
Renal	88 (21.2)	127 (21.7)	0.86
Respiratory	239 (57.6)	344 (58.7)	0.73
Cardiovascular	205 (49.4)	360 (61.4)	<0.001
Central nervous system	142 (34.2)	241 (41.1)	0.03
Coagulation	31 (7.5)	66 (11.3)	0.046
Hepatic	15 (3.6)	37 (6.3)	0.06
Treatment on day one in the ICU			
Vasoactive agents	247 (59.5)	432 (73.7)	<0.001
Mechanical ventilation	315 (75.9)	469 (80.0)	0.12
Renal replacement therapy	37 (8.9)	51 (8.7)	0.91
Glucocorticoids for septic shock	104 (25.1)	143 (24.4)	0.81

^a^Culture-negative patients were more likely to have no comorbidities than culture-positive patients (*P *= 0.001, significant after Bonferroni correction); ^b^white blood cell values available for all patients; procalcitonin values available for 266 culture-negative and 359 culture-positive patients; C-reactive protein values available for 120 culture-negative patients and 174 culture-positive patients; ^c^organ failure defined as SOFA score >2 for the specified organ. ICU, intensive care unit; COPD, chronic obstructive pulmonary disease; APACHE, Acute Physiology and Chronic Health Evaluation; SOFA, Sequential Organ Failure Assessment.

As shown in Table 2, the lungs were commoner sites of infection, while liver abscesses, biliary tract, urinary tract, soft tissue and skin infections, infective endocarditis and primary bacteremia were less common in culture-negative than in culture-positive patients.

**Table 2:** Site of infection.

Site of infection^a^	Culture-negative patients (*n *= 415)	Culture-positive patients (*n *= 586)	*P *value
Lungs	309 (74.5)	351 (59.9)	<0.001
Abdomen	41 (9.9)	78 (13.3)	0.10
Enteritis and colitis	22 (5.3)	18 (3.1)	0.08
Biliary	8 (1.9)	25 (4.3)	0.04
Peritonitis	6 (1.4)	13 (2.2)	0.38
Liver abscess	1 (0.2)	17 (2.9)	0.002
Other abscess	(0.2)	4 (0.7)	0.41
Pancreatic	1 (0.2)	1 (0.2)	1.00
Other abdominal	2 (0.5)	0 (0)	0.17
Urinary tract	17 (4.1)	71 (12.1)	<0.001
Soft tissue and skin	10 (2.4)	31 (5.3)	0.02
Nervous system	2 (0.5)	8 (1.4)	0.21
Bones and joints	2 (0.5)	8 (1.4)	0.21
Infective endocarditis	2 (0.5)	14 (2.4)	0.02
Primary bacteremia	0 (0)	12 (2.0)	0.002
Others	14 (3.4)	15 (2.6)	0.45
Unknown	18 (4.3)	29 (4.9)	0.65

^a^Some patients had more than one site of infection.

Table 3 lists the cultures performed within the two days before and the two days after ICU admission and the culture positivity rates. While more cultures were obtained from bile, liver abscesses, and soft tissue and skin in the culture-positive group than in the culture-negative group, there were no significant differences in the proportion of patients for which other cultures were performed in the two groups. Blood, urine, and endotracheal aspirate cultures were most frequently performed. A median of two blood cultures, one urine culture, and one endotracheal aspirate culture were obtained for both the culture-negative and the culture-positive patients.

**Table 3:** Cultures performed.

Type of culture^a^	Culture-negative patients (*n *= 415)	Culture-positive patients (*n *= 586)	*P *value	Rate of culture positivity (%)^b^
Blood	410 (98.8)	579 (98.8)	1.00	20.3
Urine	310 (74.7)	445 (75.9)	0.65	11.0
Endotracheal aspirate	216 (52.0)	332 (56.7)	0.15	39.4
Expectorated sputum	101 (24.3)	146 (24.9)	0.84	34.8
Bronchoalveolar lavage	24 (5.8)	32 (5.5)	0.83	51.7
Pleural fluid	12 (2.9)	14 (2.4)	0.62	14.8
Stool	43 (10.4)	74 (12.6)	0.27	4.2
Peritoneal fluid	9 (2.2)	22 (3.8)	0.15	20.6
Bile	0 (0)	6 (1.0)	0.045	83.3
Liver abscess	0 (0)	7 (1.2)	0.046	85.7
Other abscess	1 (0.2)	3 (0.5)	0.65	60.0
Soft tissue and skin	9 (2.2)	36 (6.1)	0.003	65.5
Cerebrospinal fluid	8 (1.9)	8 (1.4)	0.48	12.5
Synovial fluid	1 (0.2)	5 (0.9)	0.41	88.9
Others	6 (1.4)	14 (2.4)	0.29	56.5

^a^Cultures collected within two days before and the two days after ICU admission were included; ^b^calculated as (total number of positive cultures divided by total number of cultures performed) × 100. ICU, intensive care unit.

Table 4 features the microbiology. Gram-positive bacteria were isolated in 257 patients (25.7%) while Gram-negative bacteria were isolated in 390 patients (39.0%). Among these patients, 196 (19.6%) had only Gram-positive infections, 329 (32.9%) only Gram-negative infections, while 61 (6.1%) had mixed Gram-positive and Gram-negative infections. *Staphylococcus aureus *and *Klebsiella pneumonia *were the commonest Gram-positive and Gram-negative microorganisms respectively.

**Table 4:** Bacteria isolated.

Bacteria isolated^a^	No. of patients (% of entire cohort of patients)	
	All cultures	Bacteremia
Gram-positive	257 (25.7)	120 (12.0)
Methicillin-sensitive *Staphylococcus aureus*	82 (8.2)	39 (39.0)
Methicillin-resistant *Staphylococcus aureus*	59 (5.9)	10 (1.0)
* Streptococcus pneumoniae*	67 (6.7)	39 (3.9)
Other *Streptococcus *species	19 (1.9)	15 (1.5)
Enterococcus	28 (2.8)	16 (1.6)
Others	9 (0.9)	5 (0.5)
		
Gram-negative	390 (39.0)	213 (21.3)
* Klebsiella pneumoniae*	131 (13.1)	73 (7.3)
* Escherichia coli *	117 (11.7)	80 (8.0)
* Pseudomonas aeruginosa*	62 (6.2)	15 (1.5)
* Acinetobacter baumannii*	35 (3.5)	10 (1.0)
* Bulkholderia pseudomallei*	13 (1.3)	10 (1.0)
* Enterobacter cloacae*	10 (1.0)	4 (0.4)
* Hemophilus influenzae*	9 (0.9)	1 (0.1)
* Salmonella *species	7 (0.7)	3 (0.3)
* Citrobacter *species	6 (0.6)	3 (0.3)
* Stenotrophomonas maltophilia*	6 (0.6)	1 (0.1)
* Proteus *species	5 (0.5)	5 (0.5)
* Bacteroides fragilis*	5 (0.5)	2 (0.2)
Others	23 (2.3)	14 (1.4)

^a^More than one species of bacteria isolated in some patients.

Patient outcomes are presented in Table 5. Culture-negative patients had a shorter duration of hospital stay, and lower ICU mortality and hospital mortality (35.9% versus 44.0%, *P *= 0.01) than culture-positive patients.

**Table 5:** Outcomes.

	Culture-negative patients (*n *= 415)	Culture-positive patients (*n *= 586)	*P *value
Hospital mortality	149 (35.9)	258 (44.0)	0.01
ICU mortality	139 (33.5)	232 (39.6)	0.049
Duration of mechanical ventilation, days^a^	3.0 (1.0-7.0)	4.0 (1.0-8.0)	0.35
Duration of ICU stay, days	4.0 (2.0-8.0)	4.0 (2.0-9.0)	0.46
Duration of hospital stay, days	12.0 (7.0-21.0)	15.0 (7.0-27.0)	0.02

^a^A total of 326 culture-negative patients and 448 culture-positive patients were mechanically ventilated during the ICU stay. ICU, intensive care unit.

Table 6 details the variables associated with hospital mortality. While culture positivity was associated with higher mortality on univariable analysis, it did not feature as an independent predictor of mortality after accounting for other covariates on logistic regression analysis. The same applies to the administration of inappropriate antibiotics on the day of ICU admission. Multivariable analysis revealed the following independent predictors of mortality: age, time from hospitalization to ICU admission, lung, bone and joint infections, infective endocarditis, primary bacteremia, Acute Physiology Score, coagulation and hepatic failures, and mechanical ventilation on the day of ICU admission. The logistic regression model fitted well and there was no multicollinearity. In a separate analysis including specific microorganisms, *Pseudomonas aeruginosa *was the only pathogen which, when isolated independently, increased mortality (odds ratio (OR) 2.02, 95% CI 1.08 to 3.79, *P *= 0.03).

**Table 6:** Predictors of hospital mortality by univariable and multivariable logistic regression analyses.

Variable^a^	Univariable OR (95% CI)	*P *value	Multivariable adjusted OR (95% CI)^b^	*P *value
Demographics				
Age, per year	1.02 (1.02-1.03)	<0.001	1.03 (1.02-1.04)	<0.001
Source of admission				
Hospital floor versus ED	1.90 (1.46-2.48)	<0.001	1.34 (0.97-1.85)	0.80
Other ICU versus ED	1.38 (1.05-1.81)	0.02	1.24 (0.64-2.38)	0.53
Time of ICU admission				
Per day from hospitalization	1.06 (1.03-1.09)	<0.001	1.06 (1.03-1.09)	<0.001
Comorbid conditions				
Chronic kidney disease	1.51 (1.06-2.15)	0.02	1.29 (0.80-2.08)	0.29
Hematological malignancy	2.03 (1.01-4.09)	0.048	1.40 (0.55-3.57)	0.48
Site of infection				
Lungs	0.98 (0.75-1.27)	0.85	2.04 (1.00-4.16)	0.049
Bones and joints	5.94 (1.25-28.09)	0.03	7.31 (1.05-50.77)	0.04
Infective endocarditis	1.90 (0.70-5.13)	0.21	5.43 (1.41-20.87)	0.01
Primary bacteremia	4.46 (1.20-16.56)	0.03	8.31 (1.55-44.49)	0.01
Microbiology and antimicrobials				
Culture-positive	1.40 (1.08-1.82)	0.01	0.88 (0.59-1.31)	0.52
Inappropriate antibiotics	1.42 (1.17-1.71)	<0.001	0.86 (0.52-1.42)	0.56
Severity scores				
APS, per point	1.07 (1.05-1.09)	0.001	1.06 (1.03-1.09)	<0.001
Organ failure^c^				
Renal	1.36 (1.00-1.84)	0.049	0.99 (0.64-1.55)	0.98
Respiratory	1.31 (1.02-1.70)	0.04	0.98 (0.70-1.36)	0.90
Cardiovascular	1.71 (1.32-2.21)	<0.001	0.91 (0.57-1.44)	0.68
Central nervous system	2.17 (1.67-2.82)	<0.001	1.15 (0.80-1.65)	0.45
Coagulation	3.02 (1.95-4.68)	<0.001	2.59 (1.51-4.46)	0.001
Hepatic	4.27 (2.28-7.98)	<0.001	6.30 (2.98-13.30)	<0.001
Treatment on day 1 in the ICU				
Vasoactive agents	2.07 (1.56-2.76)	<0.001	1.42 (0.88-2.30)	0.15
Mechanical ventilation	3.17 (2.23-4.52)	<0.001	1.91 (1.21-3.02)	0.005

^a^Only variables that were significantly associated with hospital mortality on univariable or multivariable analyses are shown; ^b^Hosmer-Lemeshow test for goodness of fit for multivariable logistic regression model: χ^2 ^= 7.208, degrees of freedom = 8, *P *= 0.51; ^c^organ failure defined as Sequential Organ Failure Assessment (SOFA) score >2 for the specified organ. OR, odds ratio; CI, confidence interval; ED, emergency department; ICU, intensive care unit; APS, Acute Physiology Score, used in place of the Acute Physiology and Chronic Health Evaluation (APACHE) II score.

**Figure 1:**
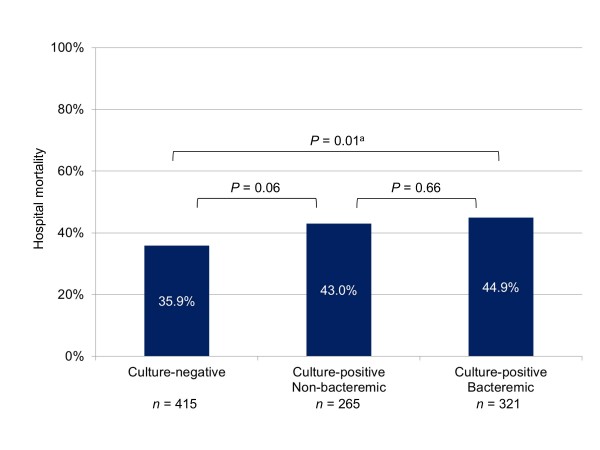
**Hospital mortality for subgroups according to cultures and bacteremia**. Overall *P *value for comparison between three subgroups was 0.03. Listed *P *values refer to comparisons between two subgroups. ^a^Significant after Bonferroni correction.

**Figure 2:**
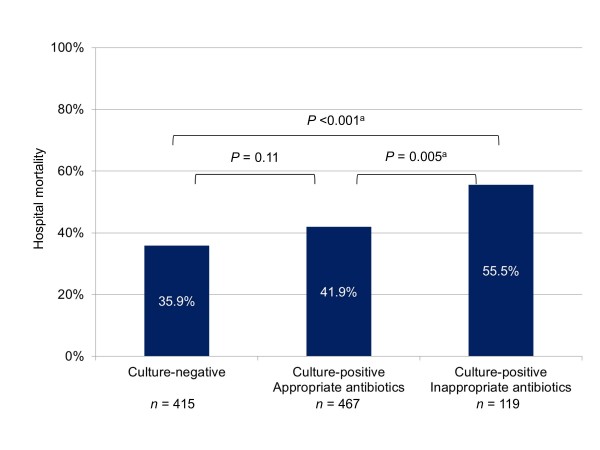
**Hospital mortality for subgroups according to cultures and receipt of appropriate antibiotics**. Overall *P *value for comparison between three subgroups was 0.005. Listed *P *values refer to comparisons between two subgroups. ^a^Significant after Bonferroni correction.

Among the culture-positive patients, 265 were nonbacteremic while 321 were bacteremic. Hospital mortality was similar in both subgroups. The nonbacteremic but culture-positive subgroup had a higher mortality rate than the culture-negative group but this was not statistically significant; the bacteremic subgroup had a significantly higher mortality rate than the culture-negative group (Figure 1). Again among culture-positive patients, 467 received appropriate antibiotics on the first day of ICU stay while 119 did not. Hospital mortality was higher in the latter subgroup. The culture-positive subgroup that received appropriate antibiotics had a higher mortality rate than the culture-negative group but this was not statistically significant; the culture-positive subgroup that did not receive appropriate antibiotics had a significantly higher mortality rate than the culture-negative group (Figure 2).

## Discussion

To the best of our knowledge, no previous study has focused on the differences between culture-negative and culture-positive severe sepsis. The main findings of this study are that patients with culture-negative sepsis had fewer comorbidities and lower severity of illness than those with culture-positive sepsis. Although culture-negative patients had a shorter hospitalization and lower ICU mortality and hospital mortality than culture-positive patients, culture positivity per se was not independently associated with mortality on multivariable analysis.

Causative microorganisms were not found in 41.5% of our patients. Multicenter studies published in the last decade found that culture-negative patients accounted for 28%, 35%, 38%, and 48% of all cases of severe sepsis in North American, Spanish, French, and Canadian ICUs, respectively [10,16-18]. The corresponding figure was 40% in the pan-European Sepsis Occurrence in Acutely Ill Patients (SOAP) study [11]. In the United States, 49% of hospitalized patients with sepsis were culture-negative [3]. Meanwhile, the Extended Prevalence of Infection in Intensive Care (EPIC) II study, which reported prevalence - and not incidence - found that 30% of all infections in ICUs worldwide were culture-negative [19]. While we found more Gram-negative pathogens, others have found a predominance of Gram-positive microorganisms [3,11].

Despite the high prevalence of culture-negative sepsis, studies which focus on the outcomes of such patients are surprisingly limited. In the 1990s, Rangel-Frausto and colleagues found a mortality of 25% among 577 patients with severe sepsis and septic shock in a teaching hospital and no difference in outcomes between culture-negative and culture-positive patients [9]. Mortality was also similar among 310 culture-negative and 742 culture-positive patients with severe sepsis in a multicenter study by Brun-Buisson and colleagues [8]. In the 2000s, a North American study led by Kumar and colleagues found similar mortality among 608 culture-negative and 1,546 culture-negative patients [10], while the pan-European SOAP study found similar ICU mortality rates (40% versus 39%) between 468 culture-negative and 454 culture-positive septic patients [11]. Our study, on the other hand, found a significantly lower hospital mortality rate for culture-negative than for culture-positive severe sepsis (35.9% versus 44.0%). Although this difference was greater for bacteremic patients than for culture-positive but nonbacteremic patients, there was no significant difference in mortality between these latter two subgroups (43.0% versus 44.9%). The reasons why we found a difference in mortality when the earlier studies did not are not immediately clear. These studies were not primarily designed for such a comparison and did not provide details on the severity of illness in these two groups of patients. In Rangel-Frausto *et al*.'s cohort, culture-negative patients were less likely to have acute kidney injury and shock, but in Brun-Buisson *et al*.'s cohort, culture-negative patients had more hypotension [8,9]. In our study, culture-negative patients were clearly less sick than their culture-positive counterparts: they had fewer comorbidities, less hemodynamic instability and organ failures, and lower APACHE II scores. After adjustment for covariates including severity of illness, identification of microorganisms was not independently associated with mortality, a finding similarly reported recently by the French OUTCOMEREA database [12].

The question that then begs to be answered is: what exactly is the cause of culture-negative sepsis? Is it merely a milder form of sepsis compared to culture-positive sepsis? While our study cannot answer these questions, its findings provide some insight into four possibilities. First, it is known that cultures lack the sensitivity to identify all bacteria. Postulated reasons include prior antibiotic exposure, sampling error, insufficient volume for blood cultures, poor transport conditions, and slow-growing or fastidious bacteria [6]. Polymerase chain reaction (PCR)-based molecular techniques may improve detection rates, and many patients with clinical sepsis are indeed PCR-positive but culture-negative [20-22]. In the PIRO system for staging sepsis, the letter 'I' refers to the nature and extent of the infection [23]. It may be hypothesized that the lower severity of culture-negative sepsis in our study was at least in part due a milder insult and lower bacterial burden [24], and correspondingly, the inability to capture the microorganisms on cultures. While it is possible that antibiotic pretreatment might have contributed to negative cultures, this is less likely for blood cultures that were usually performed before antimicrobial therapy. In addition, although we did not differentiate community-acquired from hospital-acquired infections, the short median (interquartile range (IQR)) lag time from presentation to the hospital to ICU admission of 0 days (0 to 2) suggests that most patients had the former, where the incidence of antibiotic pretreatment is likely to be lower. The letter 'P' in the PIRO system refers to predisposition [23], and given the trend toward more culture-positive sepsis among diabetic patients in our cohort, it is conceivable that diabetics are more prone to having large bacterial loads [25].

Second, it is clear from our data that certain infections are less common in culture-negative than in culture-positive patients, and vice versa. This is in part due to the nature of some infections, for example, liver abscesses are less likely to be culture-negative [26], and in part due to the diagnostic criteria for other infections, for example, the importance of blood culture positivity for primary bacteremia and in the revised Duke criteria for infective endocarditis.

Third, some of our culture-negative patients might have had nonbacterial sepsis. Fungi account for approximately 5% of cases of sepsis in ICUs and are generally more readily detected than viruses and parasites [16,17]. Our study design mandated the exclusion of such microorganisms. While we are confident that most patients with fungal sepsis were diagnosed and thus excluded, and that parasites are extremely rare in our urban setting, it is plausible that undetected viruses contributed to a significant proportion of culture-negative sepsis. Using reverse-transcription PCR assays on bronchoalveolar lavage fluid and nasopharyngeal swab specimens, Choi and colleagues recently demonstrated that severe pneumonia was due to viruses 36% of the time [27]. It is not routine clinical practice in most ICUs, including ours, to test for viruses in pneumonia, and the lungs were a commoner site of infection in culture-negative than in culture-positive patients in our study. Meanwhile, serum procalcitonin levels, which are a marker of bacterial as opposed to viral infections, were lower in our culture-negative group, again bringing up the possibility that viruses played a role [28].

Importantly, a fourth conceivable explanation for our culture-negative group is that some of the patients did not actually have sepsis. The 1992 ACCP/SCCM Consensus Conference criteria that we and many other investigators used to define sepsis [11,17,18] - as well as subsequent adaptations - are based on a composite of clinical and laboratory data and will inevitably include a heterogeneous set of diagnoses, some of which are false positives and unrelated to infections [13,14,23]. In a study by Heffner and colleagues, 32% of culture-negative patients who were initially identified as having severe sepsis in the emergency department were subsequently found to have noninfectious mimics while 16% had illnesses of indeterminate etiology [29]. Among our culture-negative patients, 74.5% had a lung infection, compared to 64% in the European SOAP cohort [11]. This might have been due to the fact that ours is a medical ICU, but one could also postulate that some of our patients had mimickers of pneumonia such as heart failure. However, because we used all available clinical information from ICU admission till discharge or death before labeling severe sepsis, including noninvasive and invasive hemodynamic monitoring where indicated, it is likely that the proportion of patients who did not actually have sepsis in our study was smaller than in Heffner and colleagues' cohort.

What are the implications of our study's findings? Early administration of broad-spectrum antibiotics is recommended by the Surviving Sepsis Campaign guidelines in an effort to improve outcomes in sepsis, culture-positive or culture-negative [5]. Kumar and colleagues showed that every hour of delay in the administration of effective antibiotics from the onset of septic shock resulted in an increase in mortality [10]. This association occurred even among culture- negative patients, for which antimicrobial therapy was deemed appropriate if they were consistent with national guidelines modified to local flora for the clinical syndrome. In contrast, similar to the majority of studies in the literature [4], our study defined the administered antibiotics to be inappropriate only if they did not match the *in vitro *susceptibility of the identified pathogens, that is, only in culture-positive patients. In so doing, we found the mortality rate to be much higher among culture-positive patients who were administered inappropriate antibiotics than culture-negative patients (55.5% versus 35.9%). There was also a trend toward higher mortality among culture-positive patients who received appropriate antibiotics (41.9%) than culture-negative patients. These findings do not invalidate the Surviving Sepsis Campaign's recommendation, especially since it is impossible to accurately predict one's culture status at presentation. On the clinical front, we echo the Surviving Sepsis Campaign guidelines' advice for cautious consideration of antimicrobial therapy using clinician judgment and available clinical information when cultures are unrevealing [5]. On the research front, we propose that it is time for more in-depth studies of culture-negative sepsis. Such investigations could come in the form of multiplex PCR amplification techniques for the quantification of bacteria, fungi, and viruses to elucidate the false-negative and true-negative rates of cultures [20,24], and interventional trials comparing algorithms to escalate, continue, narrow, or cease antibiotics coupled with a search for noninfectious etiologies when pathogens are not detected [30].

Our study has several limitations. First, it compartmentalizes sepsis into two main groups based on the identification, or lack thereof, of pathogenic microorganisms, but in reality, both groups are a mixed bag of diagnoses [31]. As discussed at length above, the culture-negative group probably included some patients with nonbacterial sepsis and patients without sepsis. Nonetheless, the incidence of culture-negative sepsis in our cohort mirrors that in multiple studies internationally [3,10,11,16-18], and this reinforces the need to better understand this real-world phenomenon. As for the culture-positive group, *Pseudomonas aeruginosa *was the only bacteria that independently increased mortality, a finding that has also been seen in European ICUs [11]. Besides, bacteremia is often seen as a harbinger of bad outcomes and thus may not be equivalent to the identification of pathogens in bodily fluids other than blood [31,32]. This notwithstanding, our analyses suggest no difference in mortality between bacteremic and nonbacteremic culture-positive sepsis. Second, because we left the performance of cultures to the discretion of the managing physicians, we are unable to rule out the fact that some patients were culture-negative simply because of inappropriate sampling. Nonetheless, given that most cultures were performed in an equal proportion of patients in the culture-negative and the culture-positive groups, this is less likely to be a significant contributing factor to our findings. Third, as ours was a single-center study conducted in a medical ICU, the details of its findings may not be extrapolated to all ICU patients. Fourth, to determine the appropriateness of antimicrobial therapy, we did not record the exact timing of administration while reviewing the antibiotics given on the first day of ICU stay. This timeline is longer than the three-hour window period from the onset of sepsis suggested by the Surviving Sepsis Campaign [5]. Nonetheless, we found in a previous study that antibiotics were administered within three hours 75.0% of the time at our ICU [33]. Fifth, as our study was observational in nature, unadjusted and hidden confounders might have influenced our results and conclusions. To illustrate, although it is unlikely that significant differences exist in the acute management of the culture-negative and the culture-positive groups, especially when microbiological results would not have been available at presentation, it should still be acknowledged that data on treatments such as fluid resuscitation are lacking. Our study also has several strengths. It is the first and largest prospective epidemiological study dedicated to the difference between culture-negative and culture-positive severe sepsis. We only included microorganisms that were deemed by the managing physicians to be pathogens as opposed to colonizers or contaminants, with reference to the International Sepsis Forum Consensus Conference guidelines [14]. To optimize accuracy, data checks were performed by the investigators.

## Conclusions

Our study identified significant differences between culture-negative and culture-positive severe sepsis, with the former group having fewer comorbidities, milder severity of illness, shorter hospitalizations, and lower ICU mortality and hospital mortality. However, after adjusting for all covariates, culture positivity did not independently predict mortality.

## Key messages

• A large proportion of patients with severe sepsis are culture-negative.

• Culture-negative patients have fewer comorbidities and lower severity of illness than culture-positive patients.

• Culture-negative patients have a shorter hospitalization than culture-positive patients.

• Although culture-negative patients have lower ICU mortality and hospital mortality than culture-positive patients, culture positivity per se is not independently associated with mortality on multivariable analysis.

• More research is required to better understand the underlying causes of culture-negative severe sepsis.

## Abbreviations

ACCP: American College of Chest Physicians; APACHE: Acute Physiology and Chronic Health Evaluation; CI: confidence interval; EPIC: Extended Prevalence of Infection in Intensive Care; ICU: intensive care unit; PCR: polymerase chain reaction; SCCM: Society of Critical Care Medicine; SOAP: Sepsis Occurrence in Acutely Ill Patients; SOFA: Sequential Organ Failure Assessment.

## Competing interests

The authors declare that they have no competing interests.

## Authors' contributions

JP, WJN, and KHL conceived the study and participated in its design and coordination. JP, WJN, KCS, CKT, TK, HFL, MYC, HSY, AT, HJK, RC, KHL, and AM participated in the data analysis and interpretation. JP drafted the manuscript. JP, WJN, KCS, CKT, TK, HFL, MYC, HSY, AT, HJK, RC, KHL, and AM participated in the revision of the manuscript, and read and approved the final manuscript.
